# Fibromyalgia-associated hyperalgesia is related to psychopathological alterations but not to gut microbiome changes

**DOI:** 10.1371/journal.pone.0274026

**Published:** 2022-09-23

**Authors:** Thomas Weber, Eva Tatzl, Karl Kashofer, Magdalena Holter, Slave Trajanoski, Andrea Berghold, Akos Heinemann, Peter Holzer, Michael Karl Herbert

**Affiliations:** 1 Department of Anesthesiology and Intensive Care Medicine, Medical University of Graz, Graz, Austria; 2 Diagnostic & Research Institute of Pathology, Medical University of Graz, Graz, Austria; 3 Institute of Medical Informatics, Statistics and Documentation, Medical University of Graz, Graz, Austria; 4 Center for Medical Research, Medical University of Graz, Graz, Austria; 5 Division of Pharmacology, Otto Loewi Research Center, Medical University of Graz, Graz, Austria; University of Illinois, UNITED STATES

## Abstract

Fibromyalgia-syndrome (FMS) is a complex disease characterized by chronic widespread pain and additional symptoms including depression, cognitive dysfunction (“fibro-fog”) and maldigestion. Our research team examined whether FMS-related pain parameters assessed by quantitative sensory testing (QST) and psychological disturbances are accompanied by alterations of the fecal microbiome. We recruited 25 patients with FMS and 26 age- and sex-matched healthy controls. Medical background, food habits, psychopathology and quality of life were assessed through questionnaires. Stool samples were analyzed by 16S rRNA gene amplification and sequencing. QST was performed according to the protocol of the German Network for Neuropathic Pain. QST showed that both lemniscal and spinothalamic afferent pathways are altered in FMS patients relative to healthy controls and that peripheral as well as central pain sensitization processes are manifest. Psychometric assessment revealed enhanced scores of depression, anxiety and stress. In contrast, neither the composition nor the alpha- and beta-diversity of the fecal microbiome was changed in FMS patients. FMS patients segregate from healthy controls in various parameters of QST and psychopathology, but not in terms of composition and diversity of the fecal microbiome. Despite consideration of several confounding factors, we conclude that the contribution of the gut microbiome to the pathophysiology of FMS is limited.

## Introduction

Patients suffering from chronic pain create a total socioeconomic burden of around 3,000 Euro per year [1]. Fibromyalgia-syndrome (FMS), a chronic pain disease with a high incidence, affects about 2–4.7% of the population worldwide [2].

Patients with FMS suffer from various symptoms, the most common one being chronic widespread pain in different body areas. Moreover, various kinds of additional symptoms can occur, such as fatigue, cognitive difficulties (“fibro-fog”), sleep disturbances, and depression. A high percentage of patients also has to deal with maldigestion, for example, irritable bowel syndrome, which is very common in FMS [2–6].

The pathophysiology of FMS appears very complex and is hardly understood. The International Association for the Study of Pain (IASP) has recently introduced the ICD-11 term of “nociplastic pain” [7] as a new designation to describe pain in the absence of actual or threatened tissue damage. FMS is classified as a disease in which nociplastic pain plays a crucial role in the pathophysiology [2, 8, 9].

It has been proposed that both genetic and environmental predisposition might play a role, including bacterial infections (e.g. Borrelia) or a stressful life event (e.g. loss of partner) [10, 11]. It is not clear whether peripheral inflammation or central nociplastic changes catalyze chronic widespread pain [12–14]. Probably due to an interplay of peripheral and central mechanisms, neuromorphological changes are brought about that trigger further symptoms. Changes at spinal and supraspinal levels can also lead to reduced endogenous pain inhibition [15]. Furthermore, significant alterations of the autonomic nervous system occur, which may explain many of the FMS-specific symptoms [8, 16]. For instance, Furlan et al. [17] showed that patients with FMS present with enhanced cardiovascular sympathetic activity, although there are currently controversies about basal levels of sympathetic activity [18]. Other studies [19] showed even lower sympathetic levels in FMS.

Recently, the gut microbiota has been considered to be another factor relevant to the pathophysiology of FMS [20, 21] Minerbi et al. [22] and Clos-Garcia et al. [23] were able to show that specific bacteria of the gut microbiome are changed in patients with FMS. The altered bacteria were mostly gram-negative and thus can shed lipopolysaccharides (LPS) that may become systemic through a leaky gut barrier. Via activation of Toll-like receptor-4 (TLR-4) on monocytes and neutrophil granulocytes, LPS can induce systemic inflammation which leads to neuronal alterations that contribute to central sensitization [20].

In the studies of Minerbi et al. [22] and Clos-Garcia et al. [23] FMS was diagnosed by a thorough clinical assessment of the patients involving a variety of questionnaires. Since self-reported pain profiles do not always match quantitative sensory testing (QST) results in FMS patients [24–26], the first objective of the current study was to investigate whether FMS-related psychopathological and QST profile changes are accompanied by alterations of the fecal microbiome. Given that FMS is linked with several psychological disturbances [27], the second objective was to examine whether FMS-related changes in psychometric scores (depression, anxiety, stress) go along with changes in microbial composition. In this way we set out to obtain more specific information on the relevance of psychopathology and the gut microbiome to the pathophysiology of FMS.

## Material and methods

This study was a single-center, case-control study, performed at the Medical University of Graz, Austria, from December 2018 until December 2019. A permission of the local ethics committee (Medical University of Graz, Austria) was obtained prior to the start of the study (registration number: EK 31–012 ex 18/19). This study was conducted according to the Declaration of Helsinki in 1964 and the current STROBE guidelines for reporting observational studies. Informed consent was obtained from all study participants.

**Fig 1:**
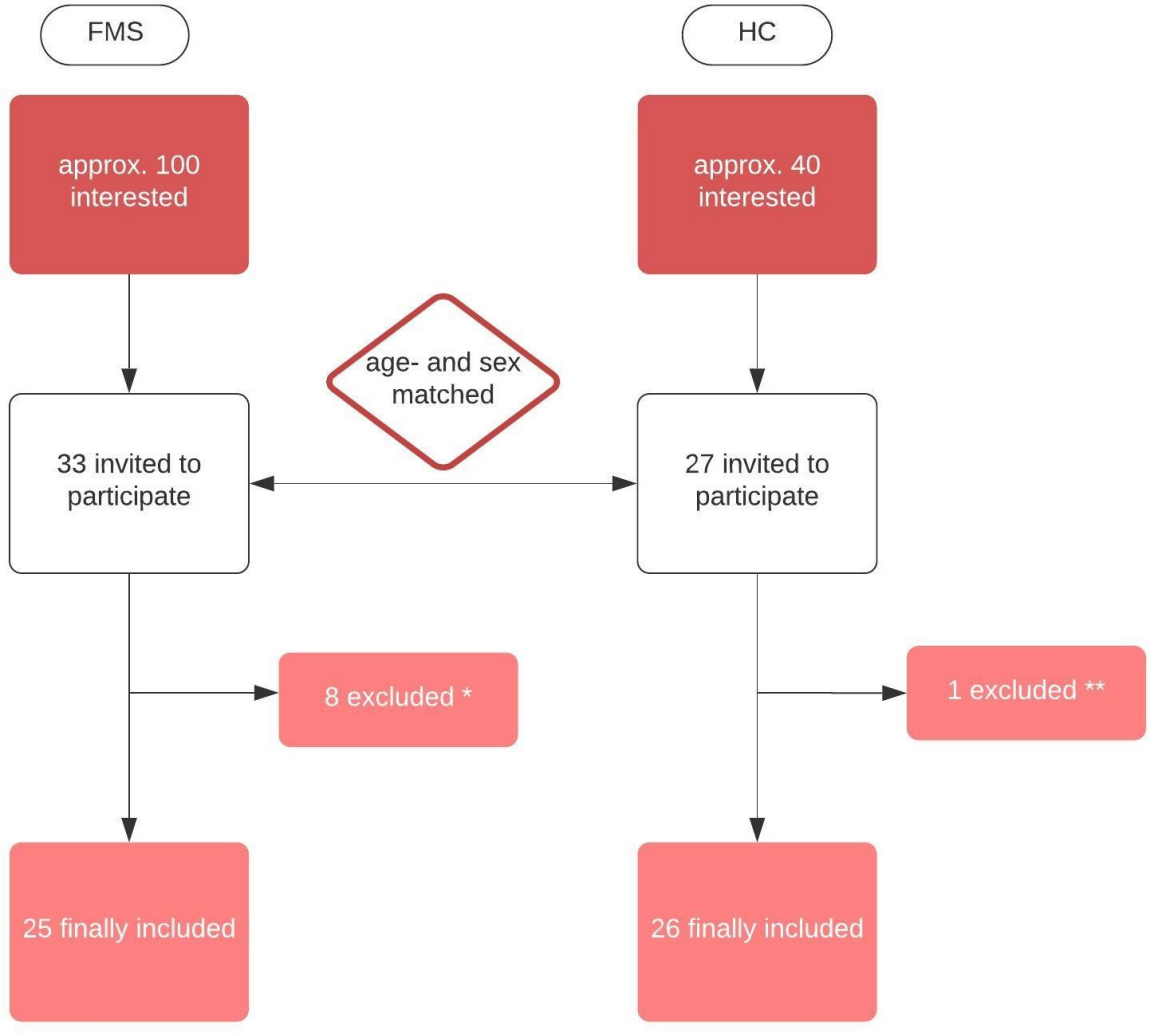
Flow chart of how many study participants were included/excluded in the study. * 8 were excluded in the FMS-group: 1 Ehlers-Danlos syndrome, 1 unclear autoimmune disease, 1 stroke in anamnesis, 1 acute severe pneumonia, 2 non-compliant, 2 lost contact. ** 1 was excluded in the healthy control (HC) group: autoimmune disease.

The research team recruited 25 patients with FMS and 26 healthy control (HC) subjects (age- and sex-matched). FMS was diagnosed according to the criteria of the American College of Rheumatology published in 2016 [28] (criteria catalogue and physical examination) and FMS symptom-severity was quantitated according to the Patient-Health-Questionnaire-15 (PHQ-15) [29]. The inclusion and exclusion procedure are illustrated in Fig 1.

FMS patients were recruited via an invitation letter at the local pain clinic. Age- and sex-matched voluntary employees at the University Hospital were asked to join the study as healthy controls. Besides signing an informed consent, one single study visit was necessary. After checking the inclusion- and exclusion criteria, all patients underwent a physical neuro-orthopedic examination to exclude reasons for musculoskeletal pain other than FMS. First, blood samples for a separate study were taken. Patients and healthy probands underwent quantitative sensory testing (QST) and were given detailed information about how to complete several questionnaires and how to handle the stool samples. Patients and healthy controls were instructed to fill the stool collection tubes (4 stool collection tubes) at home and then send them to the laboratory with a prepared, cooled package. An electronic thermometer for tracking a constant temperature (maximum temperature allowed +15°C for 24 hours) was included in each parcel. All samples were then immediately frozen at -80° C. One stool sample was needed for microbiome analysis, all others were archived.

### Demographic data

Demographic data and detailed medication anamnesis were obtained via a structured talk and documented on the German Pain Questionnaire. Furthermore, all study participants were asked to answer the validated German version of the Depression-Anxiety-Stress Scale (DASS-G) [30], the ICD-10 Symptom-Rating Brief Description (ISR-10) [31] and the Marburg Questionnaire for Quality of Life (MbFhW) [32].

Patients and HC subjects also had to complete a questionnaire about their dietary habits. This questionnaire was derived from the Food-Frequency-Questionnaire of the Robert-Koch-Institute and asks about the most important dietary habits (12 items) for a balanced diet [33].

### Microbiome analysis

Stool samples were collected in Stool Collection Tubes with DNA Stabilizer (Stratec Molecular, Berlin, Germany) and then frozen at -80°C. As investigated by Invitek Molecular©, the preserving fluid (DNA stabilizer) has no effect on the microbiome analysis of stool samples [34]. Bacterial DNA was extracted with the Maxwell RSC Blood DNA Kit (Promega, Mannheim, Germany) according to the manufacturer’s instructions with slight modifications for stool samples. The stool samples were homogenized with lysis buffer on a MagNA Lyser Instrument using MagNA Lyser Green Beads (Roche Diagnostics GmbH, Mannheim, Germany). The samples were then treated with 2.5 mg/ml lysozyme (Roth GmbH, Karlsruhe, Germany) for 30 min at 37°C followed by digestion with 1 mg/ml proteinase K for 60 min at 56°C. The enzyme was inactivated at 95°C for 10 min. For the DNA isolation in the Maxwell RSC, 600 μl of lysate was taken. The concentration of DNA was determined by Picogreen fluorescence. Then, the variable V4 region of the bacterial 16S rRNA gene was amplified using the Mastermix 16s Complete PCR Kit (Molzym, Bremen, Germany) according to the manufacturer’s instructions from 20 ng DNA using oligonucleotide primers 16s_515_fwd: TGCCAGCAGCCGCGGTAA and 16s_806_rev: GGACTACCAGGGTATCTAAT. Afterwards PCR products were subjected to agarose gel electrophoresis and the band of the expected length (350 nt) was excised from the gel and purified using the QiaQick (Qiagen, Hilden, Germany) gel extraction system. The amplicon DNA concentration was measured by Picogreen fluorescence.

Amplicons from 30 samples were pooled equimolarly and subjected to emulsion PCR in the Ion Chef™ Instrument according to the manufacturer’s protocols using the Ion 400BP workflow and the Ion 530™ Chip Kit. Sequencing reactions were performed on the Ion GeneStudio S5 System running for 1000 flows (all reagents from Thermo Fisher Scientific, MA, USA). The sequence files were analyzed with GALAXY using the QIIME 2019.7 workflow [35–39].

### Quantitative sensory testing (QST)

QST was carried out according to the protocol of the German Network for the Treatment of Neuropathic Pain (DFNS) [40] on the non-dominant hand. To measure heat and cold sensory levels and heat and cold pain threshold, PATHWAY (Medoc, Ramat Yishai, Israel) was used. Vibration detection threshold was measured with VSA-3000 (Medoc, Ramat Yishai, Israel), pressure detection threshold with Force dial FDK/FDN series (Wagner, Greenwich, CT, USA), mechanical-tactile threshold with von Frey filaments (MARSTOCKnervtest, Marburg, Germany), and dynamic-mechanical sensory threshold with Pinprick (MRC Systems, Heidelberg, Germany). Patients and healthy controls were always tested by the same examiner at the same room temperature, given that the ambient temperature has an influence on pain sensitivity in FMS [41].

### Statistical analysis

The demographic data were described by mean, standard deviation, median, minimum and maximum as appropriate for continuous variables, whereas categorical variables were described by absolute and relative frequencies. The demographic data and nutrition scores of FMS patients and healthy controls were compared by t-test and Fishers exact test.

Statistical analysis was performed with SPSS version 27, IBM®, p<0.05 was defined as statistically significant. We used G*Power [42] for calculating the number of study participants in FMS and healthy controls. A sample size of 26 in each group will have 80% power to detect an effect size of 0.8 using a two group t-test with a 5% two-sided significance level.

For analysis of the human gut microbiome, the Galaxy web platform (http://galaxy.medunigraz.at) [38] was used. Operational taxonomic units (OTUs) were displayed as OTU tables created with QIIME2 implementation in Galaxy (Version 2019.7) DADA2-based workflow and visualized as principal coordinates analysis (PCoA) plots, and according bar charts were also generated with QIIME2. For the taxonomic classification we used SILVA rRNA database ver 132. Significant differences between FMS and HC (p<0.01) were analyzed using the Adonis test, and significant differences in individual bacterial strains were calculated by the Kruskal-Wallis test. Canonical correspondence analysis (CCA) and differential taxa abundance analysis with DeSeq2 were performed in R® according to standard protocols [43].

Raw data of QST underwent further data preparation. After checking the standard distribution with the Shapiro-Wilk test (values above p>0.05 were considered as a normal curve of distribution), a small constant (+0.1; Bartlett-Procedure) was added according to Rolke et al. [40] where applicable. Standard logarithmic transformation (ln) was done for all values except paradoxical heat sensations, cold and heat pain threshold and vibration detection threshold. Parameters were then compared with ANOVA or t-test. Further data preparation for interpretation of gain or loss of function made it necessary to z-transform all values. For FMS, z-scores (z-score = (x_single participant_−mean_norms_)/SD_norms_) were calculated and then displayed with Excel 2016, Microsoft®. Values above 0 indicate a gain of function, values below 0 a loss of function [40].

## Results

### Demographic data and general clinical assessment

All results regarding demographic data are displayed in Table 1.

**Table 1:** Demographic data (age, sex, BMI, smoking status) and dietary, medication and psychometric profiles of patients with FMS vs healthy controls.

	FMS (N = 25)	Healthy controls (N = 26)	p
**Demographics**			
Female, n (%)	22 (88)	21 (81)	0.69
Age in years, mean (SD)	49.8 ±8.6	50.0 ±8.0	0.91
BMI kg/m², mean (SD)	25.6 ±5.6	23.8 ±4.0	0.11
Smoker, n (%)	6 (24)	-	<0.01
**Diet**			
Omnivore, n (%)	17 (68)	21 (84)	
Vegetarian, n (%)	7 (28)	5 (16)	
Vegan, n (%)	1 (4)	-	
Nutrition Score, mean (SD)	5.52 ±1.39	4.32 ±1.49	0.78
**Medications**			
NSAID, n (%)	17 (68)	8 (31)	0.17
Antidepressant, n (%)	9 (36)	2 (8)	<0.01
Antihypertensive Drugs, n (%)	5 (20)	2 (8)	0.12
PPI, n (%)	6 (24)	2 (8)	0.01
Antibiotics, n (%)	3 (12)	-	0.20
THC/CBD, n (%)	10 (40)	-	<0.01
**Psychometric questionnaires**			
Depression, Median (IQR)	6.90 (8.00)	1.25 (1.00)	<0.01
Anxiety, Median (IQR)	7.39 (5.00)	1.25 (1.75)	<0.01
Stress, Median (IQR)	11.20 (8.00)	2.45 (3.00)	<0.01
Symptom Rating, Median (IQR)	1.06 (0.84)	0.30 (0.31)	<0.01
Quality of Life, Median (IQR)	16.80 (13.00)	37.64 (6.00)	<0.01

Diet: Number of patients with FMS vs healthy controls under an omnivore, vegetarian or vegan diet. Adapted nutrition score (by the German Food Frequency Score) in FMS patients vs healthy controls. Medication taken by the study participants on a regular basis for at least 3 months before the study visit. Psychometric scores assessed by DASS (Depression-Anxiety-Stress-Score) with sub results of D (Depression), A (Anxiety) and S (Stress), ISR-10 (ICD-10-Symptom-Rating) and MbFhW (Marburg Questionnaire for Quality of Life) in FMS patients vs healthy controls. The values shown are means, with SD or IQR given in parenthesis. Fishers exact test was used to calculate the p-values shown above.

Abbreviations: BMI–body mass index, NSAID–non-steroidal anti-inflammatory drug, PPI–proton pump inhibitor, THC–tetrahydrocannabinol, CBD–cannabidiol

There were no significant differences in age, sex and body mass index (BMI) between FMS and HC individuals. However, significantly (p<0.01) more patients with FMS were smokers than HC subjects. Thirteen patients presented with mild FMS (<14 points in PHQ-15) while 12 patients were classified as suffering from severe FMS (>15 points in PHQ-15). Detailed anamnesis included the presence of gastrointestinal disorders as asked for in the German Pain Questionnaire. In the FMS group, 11 patients reported dyspepsia and 7 esophageal reflux, while all healthy controls denied any disorders of the gastrointestinal tract. Another key point in FMS diagnosis was the assessment of the psychopathological profile. All pertinent questionnaires showed a significant difference between the two groups. The absolute scores were clinically relevant and confirmed that the two groups differ with regard to depression, anxiety and stress. Regarding dietary habits, neither the distribution of an omnivore diet, vegetarian diet or vegan diet among FMS patients vs. HC subjects showed significant differences, nor the calculated nutrition score between healthy controls and FMS patients. Furthermore, there were no significant group differences between the three nutritional components (source of carbohydrates, protein and fat; data not shown). All study participants completed a questionnaire regarding medication intake within the last 3 months. The results show that the use of various medications was more prevalent in FMS patients than HC subjects.

### Microbiome analysis

Stool samples were sequenced with a total of 3,491,933 reads and an average of 68,838 ± 73,362 reads per sample. Detailed analysis showed that the Shannon alpha-diversity, Evenness vector, Faith’s phylogenetic diversity, observed OTUs, inverse Simpson relation, ace and chao1 (Table 2) did not differ between the two groups.

**Table 2:** Alpha-diversity statistics for the microbiome in FMS patients vs. healthy controls.

	FMS	Healthy controls	p
OTUs	194.85 (42.98)	197.99 (49.69)	0.81
Faith-PD	16.07 (2.71)	16.13 (2.98)	0.95
Evenness vector	0.73 (0.05)	0.73 (0.05)	0.96
Shannon	5.58 (0.56)	5.58 (0.56)	0.92
Inverse Simpson	0.15 (0.04)	0.14 (0.05)	0.79
ace	212.46 (139.90)	185.53 (41.58)	0.18
chao1	183.37 (50.84)	187.96 (44.04)	0.47

The values shown are means, with SD given in parenthesis. P-values were calculated with Kruskal-Wallis Test.

Abbreviations: OTU–operational taxonomic unit, Faith PD–Faith´s phylogenetic diversity, ace–abundance-based coverage estimators, chao1 –Chao1index

**Fig 2:**
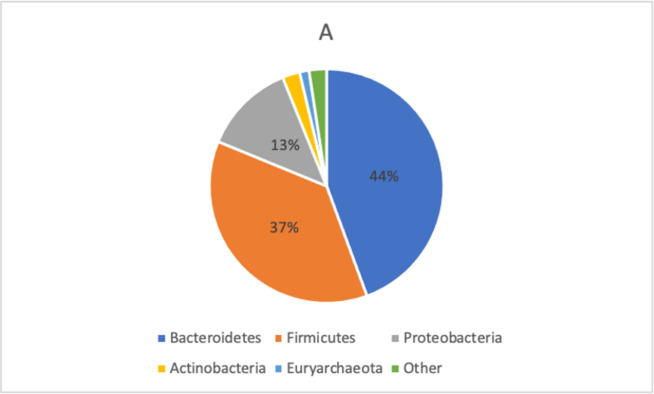
Taxonomic composition of bacterial phyla in FMS patients. In FMS, mostly dominant were Bacteroidetes (44%), followed by Firmicutes (37%) and Proteobacteria (13%). All phyla were tested for differences with DeSeq2 (GMPR normalization) which did not reveal any significant results. Abbreviations: gmpr–geometric mean of pairwise ratios.

**Fig 3:**
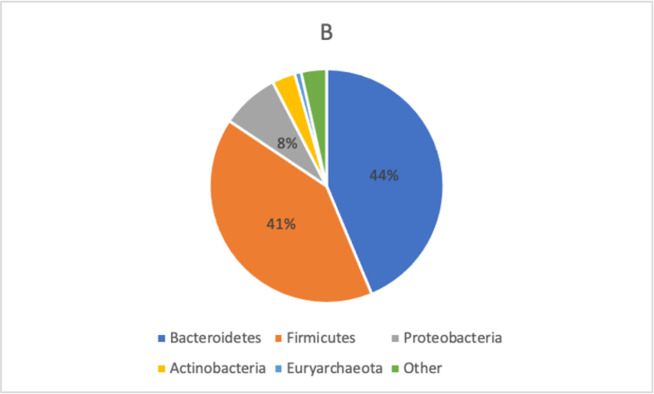
Taxonomic composition of bacterial phyla in healthy controls. For healthy controls, the predominant phyla were Bacteroidetes (44%), Firmicutes (41%) and Proteobacteria (8%). All phyla were tested for differences with DeSeq2 (GMPR normalization) which did not reveal any significant results. Abbreviations: gmpr–geometric mean of pairwise ratios.

The distribution of bacterial taxa in FMS patients and healthy controls is displayed in Figs 2 and 3.

Although subtle differences in the distribution of bacterial taxa between the two groups were observed, these differences were not statistically significant (Table 3).

**Table 3:** Statistical analysis of microbial beta-diversity in FMS patients vs healthy controls.

	FMS	Healthy Controls	p
Bray-Curtis	0.99±0.01	0.99±0.02	0.238
Jaccard distance	1.0±0.01	0.99±0.02	0.357
Unweighted unifracture	0.15±0.03	0.15±0.03	0.259
Weighted unifracture	0.53±0.11	0.52±0.12	0.425

The values shown are means with SD. ANOVA was used to calculate p-values.

**Fig 4:**
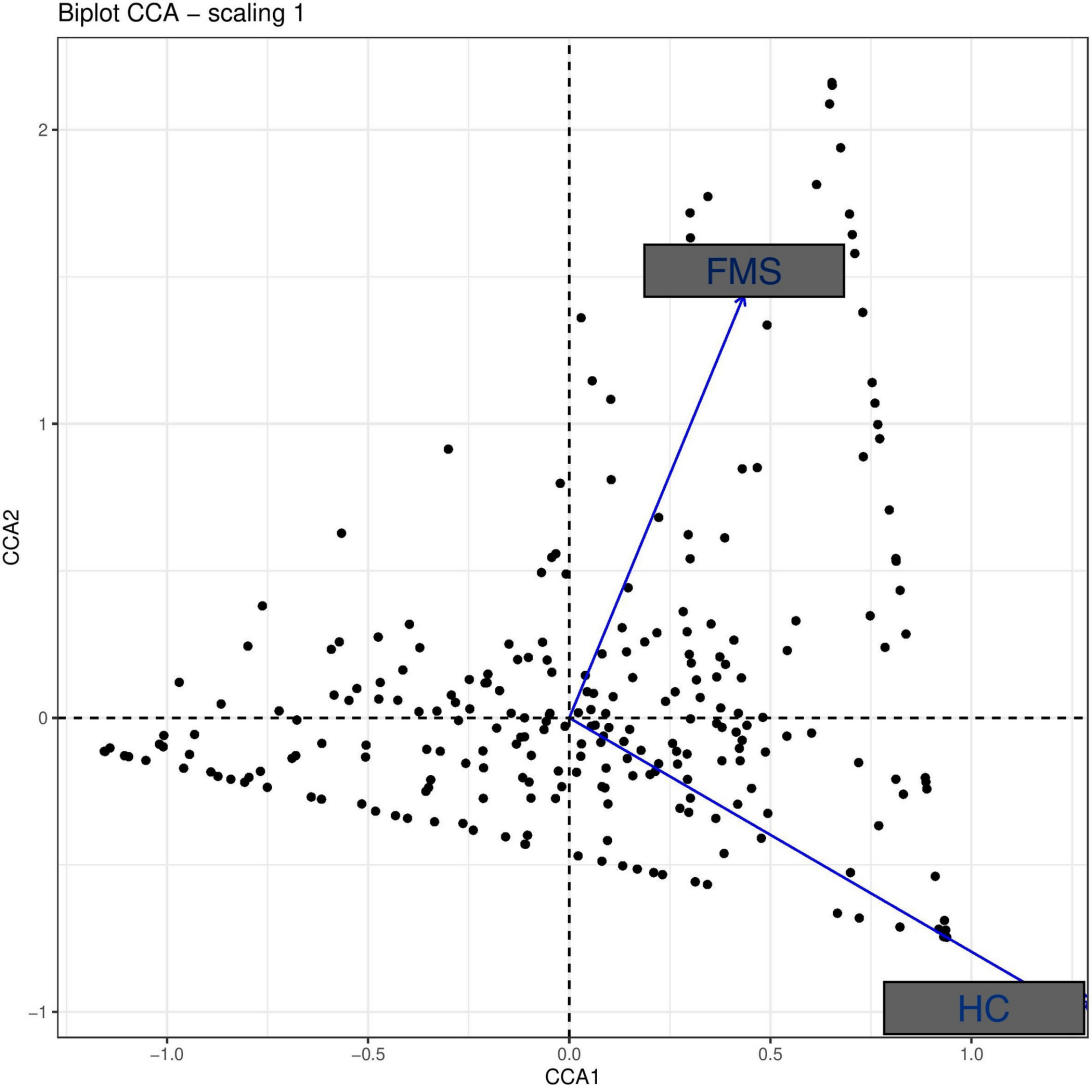
Canonical correspondence analysis (CCA) of the microbiota. CCA analysis of microbiome in FMS patients and healthy controls (HC) failed to reveal a distinct “cluster” to each group.

Canonical correspondence analysis (CCA) of the bacterial composition in FMS patients and healthy controls was also unable to disclose a significant difference (Fig 4). Furthermore, differential taxa abundance analysis with DESeq2 likewise failed to reveal clinically relevant significant differences of the bacterial strains (see S1 File).

### Quantitative sensory testing (QST)

**Fig 5:**
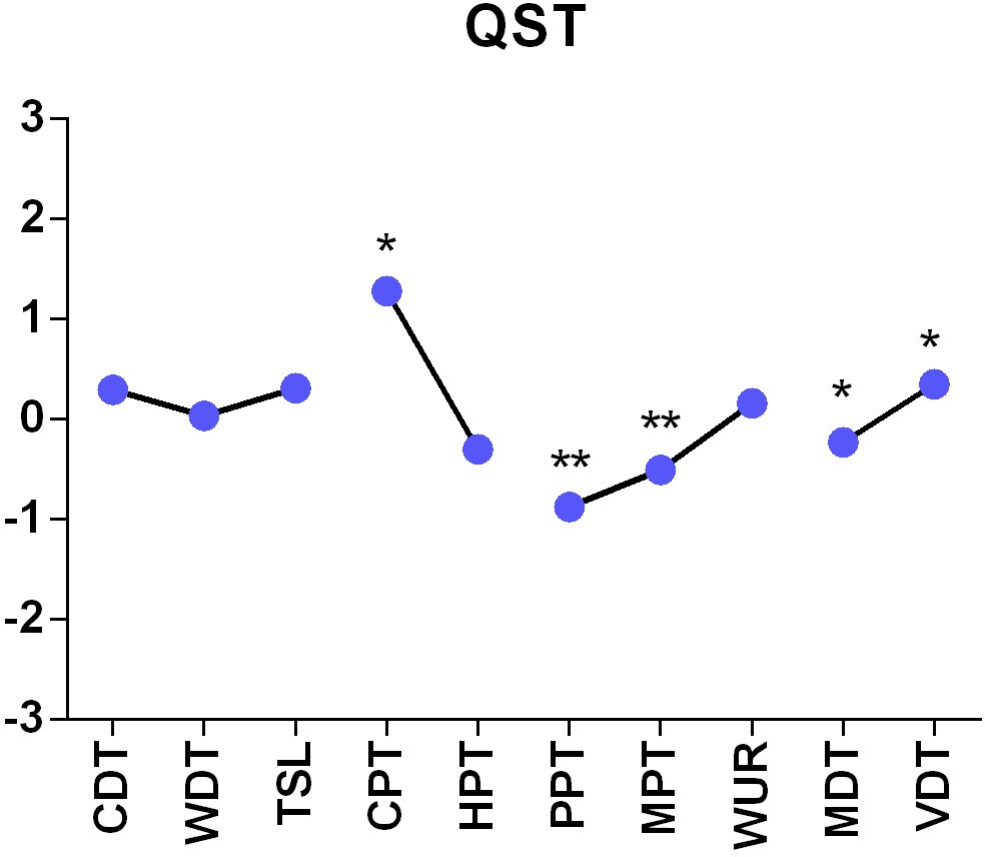
Z-adjusted values of the variables measured in quantitative sensory testing in FMS patients relative to healthy controls. * p<0.05, **p<0.001; we used ANOVA for statistical testing. CDT = cold detection threshold, WDT = warm detection threshold, CPT = cold pain threshold, HPT = heat pain threshold, TSL = temperature sensory limen, MDT = mechanical detection threshold, MPT = mechanical pain threshold, WUR = wind up ratio, PPT = pressure pain threshold, VDT = vibration detection threshold.

**Fig 6:**
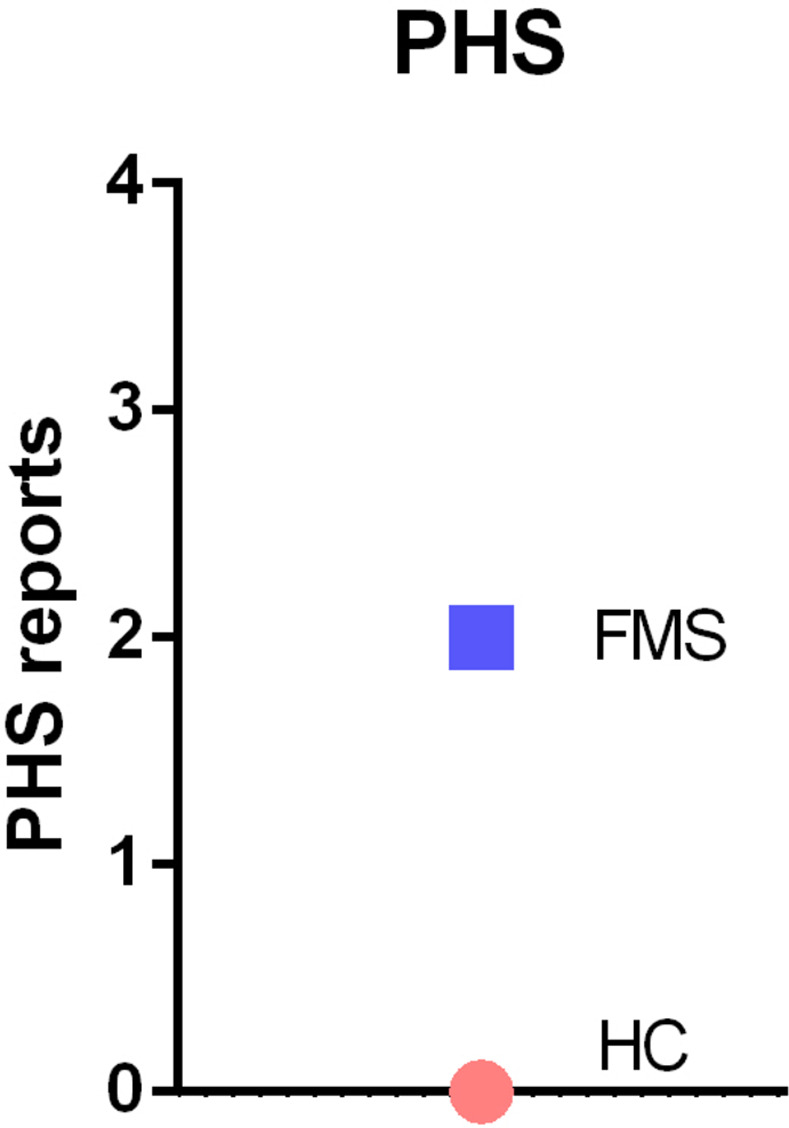
Z-adjusted values of paradoxical heat sensations measured in quantitative sensory testing in FMS patients relative to healthy controls. We used ANOVA for statistical testing. PHS = paradoxical heat sensations.

**Fig 7:**
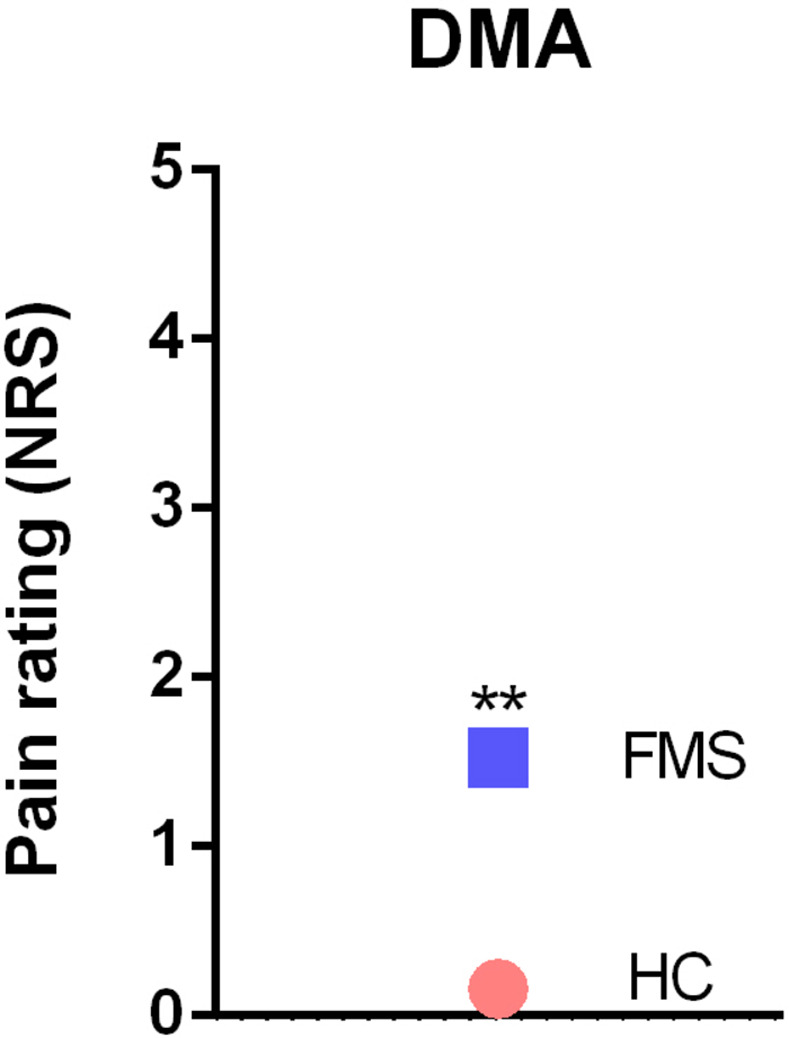
Z-adjusted values of dynamic mechanic allodynia measured in quantitative sensory testing in FMS patients relative to healthy controls. **p<0.001; we used ANOVA for statistical testing. DMA = dynamic mechanical allodynia.

QST was able to disclose significant differences in the cold pain threshold (p<0.05), pressure pain threshold (p<0.001), vibration detection threshold (p<0.05), mechanical detection threshold (p<0.05), mechanical pain threshold (p<0.001) and dynamic mechanical allodynia (p<0.001) between FMS patients and HC subjects (Table 4). To set all variables measured in the two populations in relation, we calculated z-values and displayed them in Figs 5–7.

**Table 4:** Results of quantitative sensory testing of FMS patients vs healthy controls (HC, raw data).

QST parameter	FMS	HC	p
CDT °C	30.82 (0.77)	30.59 (0.76)	0.08
WDT °C	34.26 (1.50)	34.22 (1.17)	0.85
CPT °C*	18.15 (8.91)	9.09 (6.71)	0.001
HPT °C	41.06 (3.46)	42.02 (3.15)	0.51
TSL °C	33.44 (1.41)	33.06 (1.21)	0.44
MDT mN	0.99 (0.69)	1.16 (0.74)	0.48
MPT mN*	32.94 (36.28)	49.82 (51.63)	0.04
MPS *	5.78 (8.95)	1.10 (2.15)	0.001
DMA *	1.52 (1.36)	0.16 (1.29)	0.002
PHS n	0.12 (0.44)	0 (0)	0.15
WUR	3.45 (3.38)	2.80 (1.17)	0.91
PPT kPa*	2.08 (0.66)	2.91 (1.13)	0.003
VDT μm*	0.82 (0.74)	0.66 (0.47)	0.04

The values shown are means, with SD given in parenthesis;

* indicates a significant p-value, ANOVA was used as a statistical test.

Abbreviations: CDT = cold detection threshold, WDT = warm detection threshold, CPT = cold pain threshold, HPT = heat pain threshold, TSL = temperature sensory limen, MDT = mechanical detection threshold, MPT = mechanical pain threshold, MPS = mechanical pain sensitivity, DMA = dynamic mechanical allodynia, PHS = paradoxical heat sensations, WUR = wind up ratio, PPT = pressure pain threshold, VDT = vibration detection threshold

## Discussion

The results of this study show that patients suffering from FMS present with multiple changes in pain modalities as assessed by QST which go along with enhanced scores of stress, depression and anxiety. These findings attest to complex alterations in pain sensitivity in the periphery and pain processing in the central nervous system that appear to be interrelated with disturbances of emotion, affect and stress resilience. The present results failed, however, to confirm recent reports that changes in the gut microbiota may contribute to the symptoms of FMS. We were unable to find any significant differences in the composition and diversity of the gut microbiota as analyzed in stool samples of FMS patients vs healthy controls.

The results of QST imply that both peripheral and central sensitization processes contribute to the symptoms of FMS. The lower thresholds (see Table 4) for thermal and mechanical stimuli (CPT, MDT, MPT) together with allodynia (DMA) prove the contribution of unmyelinated C-fibers and Aβ-fibers to pain sensitization. Üçeyler et al. [13] suggested that a small fiber neuropathy might contribute to chronic widespread pain in FMS. We were able to show that sensory conduction by Aβ- fibers in the lemniscal pain pathway (fine touch, vibration, proprioception) is altered in FMS. Especially dynamic mechanical allodynia and vibration detection threshold (DMA, VDT) show positive z-values (Fig 4). As a conclusion, peripheral sensitization seems to make an important contribution to chronic widespread pain, but based on these findings it stays unknown whether additional central sensitization develops as a result of altered peripheral input or vice-versa [44].

Central pain sensitization is one key domain in FMS. Furthermore, the psychopathological profile of FMS patients may contribute to the symptoms of FMS [27]. The psychopathological profile may, on the one hand, contribute to enhanced pain sensation while, on the other hand, chronic pain may give rise to anxiety, depression and stress [45]. Our data clearly show that psychological stress but also the anxiety, depression and symptom severity scores are elevated in patients with FMS vs. HC subjects (DASS-S 11.2 vs. 2.45) and their quality of life is impaired. Stress has been reported to impair central pain control in FMS patients [46]. In addition, enhanced sympathetic nerve activity has an adverse impact on pain intensity in FMS patients [8, 17]. Our findings are in line with reports that psychopathological alterations of the anxious-depressive-type are highly prevalent in FMS patients and that these psychological disturbances may lower the capacity to cope with the disease [27].

Given that the gut microbiota has been proposed to impact on pain processing in the brain [20, 47, 48] and this has also been reported for FMS [22, 23], we set out to investigate whether distinct changes disclosed by QST and psychometric evaluation could be associated to distinct changes in gut microbiome composition. However, our analysis of FMS patients vs. healthy controls failed to disclose any significant alterations in the profile of the fecal microbiome as assessed by Shannon alpha-diversity and various beta-diversity indices (Bray-Curtis, observed OTUs, evenness vector, Faith’s phylogenetic diversity, Jaccard distance, unweighted and weighted unifracture, canonical correspondence analysis, and differential gene expression analysis). This finding is at variance with the reports of Minerbi et al. [22] and Clos-Garcia et al. [23]. While in the study of Minerbi et al. [22] the overall population structure and diversity of the microbiome in FMS patients was relatively similar to those in healthy reference subjects, the study Clos-Garcia et al. [23] revealed that larger cohorts of FMS patients are needed to detect FMS-related differences especially in the beta-diversity of the fecal microbiome and to identify bacterial taxa that are up- or down-regulated. Given the limited number of subjects that could be recruited, the present study might not have the power to disclose subtle FMS-associated alterations of the fecal microbiome as the number of study participants (25 FMS vs 26 HC) was relatively small with a drop-out rate around 20%. In addition, the stool samples of two FMS patients could not be used for microbiome analysis due to low quality of the PCR results. Moreover, 3 patients included in the study reported regular antibiotic intake within the last three months. Although they withdrew from the antibiotic treatment at least 10 days before fecal sampling, an impact on the microbial community cannot be excluded. Unattended lifestyle factors such as exercise may also have had a relevant impact on the gut microbiome.

Microbiome analysis was restricted to the variable V4 region, which is a methodological limitation of the study. Examination of both the variable V3 and V4 regions in a larger cohort of FMS patients might increase the sensitivity and resolution of analysis and thus be more likely to reveal possible change in the microbial community. Apart from these limitations of the current study, there are several other confounding factors that impact on the variability of the fecal microbiome, including selection of the recruits, sex, dietary habits, sample collection and analysis methods. Given that the gut microbiome is under the influence of many internal and external factors, disease-related factors are also relevant, especially in patients suffering from chronic pain such as FMS. For instance, the psychopathology of the FMS patients studied here could have an impact, given that various neuropsychiatric disorders including depression are associated with a disturbance of the gut microbiome [49, 50]. The DASS-D, DASS-A and DASS-S scores in FMS patients were all significantly higher than those in healthy controls which is likely to be associated with increased activity in the autonomic nervous system and the hypothalamic-pituitary-adrenal axis. Another issue to be considered is that the patients were allowed to take routine medication up to the study visit. It is known that, apart from antibiotics, many drugs including NSAIDs [51], antidepressant drugs [52] and proton pump inhibitors [53] significantly alter the profile of the gastrointestinal microbiome. Furthermore, antidepressant drugs and analgesic medicine are well known to influence sensitive parameter in QST (e.g. CPT). Dietary habits are among the major determinants of microbial structure and diversity in the gut [54], but this factor may be of minor relevance to the present study because dietary habits and nutrition scores were similar in FMS patients and healthy controls. With these considerations in, it is reasonable to suspect that FMS-related alterations in the fecal microbiome may be obscured by the influence which psychopathology, medication and/or diet have on the microbiome. This contention is in line with the conclusion of Erdrich et al. [55] that the relationship between the gut microbiome and the pathophysiology of FMS remains a largely underexplored area.

In summary we conclude that, in the FMS patients studied here, the changes in pain sensing and processing are related to significant psychometric alterations that cannot be attributed to alterations in the fecal microbiome composition. However, the validity of this statement with regard to the involvement of the gut microbiome is limited by the relatively small number of study participants.

## Supporting information

S1 File (XLSX)
