# Correction: Fibromyalgia-associated hyperalgesia is related to psychopathological alterations but not to gut microbiome changes

**DOI:** 10.1371/journal.pone.0308829

**Published:** 2024-08-08

**Authors:** Thomas Weber, Eva Tatzl, Karl Kashofer, Magdalena Holter, Slave Trajanoski, Andrea Berghold, Akos Heinemann, Peter Holzer, Michael Karl Herbert

The Data Availability statement for this paper is incorrect. The correct statement is: All relevant data are available on Zenodo: 10.5281/zenodo.7149826.
